# Electrocardiography versus Echocardiography in Severe Aortic Stenosis with the Consideration of Coexistent Coronary Artery Disease

**DOI:** 10.3390/jcm13041013

**Published:** 2024-02-09

**Authors:** Michał Chyrchel, Wojciech Siłka, Mateusz Wylaź, Wiktor Wójcik, Andrzej Surdacki

**Affiliations:** 1 Second Department of Cardiology, Institute of Cardiology, Faculty of Medicine, Jagiellonian University Medical College, 30-688 Cracow, Poland; mchyrchel@gmail.com (M.C.); andrzej.surdacki@uj.edu.pl (A.S.); 2 Department of Cardiology and Cardiovascular Interventions, University Hospital, 30-688 Cracow, Poland; 3 Students’ Scientific Group, Second Department of Cardiology, Jagiellonian University Medical College, 30-688 Cracow, Poland; mateuszl.wylaz@student.uj.edu.pl (M.W.); wiktor.aleksander.wojcik@student.uj.edu.pl (W.W.)

**Keywords:** left ventricular hypertrophy, severe aortic stenosis, coronary artery disease

## Abstract

**(1) Background:** Coexistent coronary artery disease (CAD) might influence the ability of electrocardiogram (ECG) to identify echocardiographic left ventricular hypertrophy (ECHO-LVH) in patients with aortic stenosis (AS). We aimed to assess the relation between ECG–LVH (by the Sokolov–Lyon or Cornell criteria) and ECHO-LVH considering coexistent CAD. **(2) Methods:** We retrospectively analyzed the medical records of 74 patients (36 males) with severe AS who were hospitalized in the University Hospital in Cracow from 2021 to 2022. **(3) Results:** ECHO-LVH was present in 49 (66%) patients, whereas 35 (47.3%) patients had ECG-LVH. There was no difference between the rate of ECG-LVH in patients with vs. without ECHO-LVH. Single-vessel and multi-vessel CAD were diagnosed by invasive coronary angiography in 18% and 11% of patients, respectively. The sensitivity of the classical ECG-LVH criteria with regard to ECHO-LVH was low, reaching at best 41% for the Sokolov–Lyon and Cornell criteria. The results were similar and lacked a pattern when considering patients without significant stenosis, with single- and multi-vessel disease separately. Correlations between the left ventricular mass index and ECG-derived parameters were weak and present solely for the Lewis index (r = 0.31), R wave’s amplitude >1.1 mV in aVL (r = 0.36), as well as the Cornell (r = 0.32) and Sokolov–Lyon (r = 0.31) voltage criteria (*p* < 0.01). The presence, location of stenoses, and CAD extent were not associated with the presence of either ECHO-LVH or ECG-LVH, irrespective of individual ECG-LVH criteria. **(4) Conclusions:** The sensitivity of classical ECG criteria for echocardiographic LVH in severe AS is low, regardless of coexistent CAD or its angiographic extent.

## 1. Introduction

Aortic stenosis (AS) stands as an increasingly prevailing acquired valve abnormality in both North America and Europe, impacting nearly 5% of the older demographic [1,2]. This pattern is on the rise, partially due to the enhanced longevity and improved availability of medical services [3,4,5]. Progression to severe AS is associated with a poor prognosis and necessitates urgent interventional treatment using surgical aortic valve replacement (SAVR) or transcatheter aortic valve implantation (TAVI).

AS eventually leads to left ventricular hypertrophy (LVH), which is commonly regarded as a compensatory response to a chronic outflow obstruction [6]. A great quantity of heretofore published studies have reported that LVH assessment via electrocardiogram (ECG) has insufficient sensitivity, rarely exceeding 50% [7]. However, LVH changes in ECG were revealed to correlate with multiple other echocardiographic parameters, including the aortic valve area (AVA) and maximal transaortic pressure gradient (Pmax) [8]. What is more, it was observed that certain ECG tracings may feature as independent predictors of unfavorable outcomes, such as myocardial fibrosis and sudden cardiac death [9,10].

The feasibility of LVH assessment using ECG varies among patients depending on their clinical characteristics. There is an unclear impact on ECG-LVH from coronary artery disease (CAD), which might be present in up to 50% of patients with severe AS [11]. It might be the case that the relation between ECG- and echocardiographic LVH (ECHO-LVH) differs when stratifying patients according to the obstructive CAD advancement. Exploring possible trends in the varying concordance between traditional ECG and ECHO in LVH detection might aid in fully understanding the genesis of ECG changes occurring in anatomical and electrical LVH. However, since there is a great paucity of data regarding hemodynamic status, in this study, we aimed to assess the diagnostic test characteristics of ECG-LVH criteria for LVH detection with a consideration of the advancement of CAD. Furthermore, we sought to evaluate factors that are associated with both ECG- and ECHO-LVH.

## 2. Materials and Methods

### 2.1. Study Design and Population

In this retrospective analysis, we included 74 patients with severe AS (defined below), who were hospitalized in the Department of Cardiology and Cardiovascular Interventions at University Hospital in Krakow between 2021 and 2022. The exclusion criteria were as follows: other significant valvular diseases, left or right bundle-branch block, uncontrolled hypertension during echocardiographic examination, and lack of data regarding ECG tracings and echocardiographic recordings. 

Clinical and demographic characteristics were compared in two groups: patients with ECHO-LVH (N = 49) and those without ECHO criteria being matched (N = 22). 

To examine the diagnostic test characteristics of all recorded ECG tracings for ECHO-LVH recognition in the light of CAD advancement, patients were allocated into 3 groups: (i) without significant stenosis N = 46), (ii) with single-vessel disease (SVD) (N = 13), and (iii) with multi-vessel disease (MVD) (N = 8). 

The bioethics committee of our university approved the study design (No. 118.6120.04.2023). The study was conducted in line with the Declaration of Helsinki. 

### 2.2. ECG and Echocardiographic Measurements

According to European guidelines, severe AS is confirmed by an aortic peak velocity exceeding 4 m/s, a mean gradient surpassing 40 mmHg, and an AVA measuring less than 1.0 cm^2^ [12]. ECHO-LVH is defined as a left ventricular mass index (LVMI) exceeding 95 g/m^2^ in women and 115 g/m^2^ in men [13]. ECG recordings were analyzed to extract multiple criteria for LVH recognition. Nevertheless, ECG-dependent LVH was confirmed only when Sokolov–Lyon or Cornell voltage criterium was matched, as their reliability is the best supported. The former was regarded to be present if the sum of the S wave in V1 and R wave in V5/6 exceeded 3.5 mV [14]. In the Cornell voltage criteria, ECG-LVH was confirmed when the sum of the R wave’s amplitude in aVL + S wave’s amplitude in V3 exceeded 2.8 mV in men and 2.0 mV in women [15].

### 2.3. Statistical Analysis

Continuous variables are expressed as mean (±standard deviation) or median [first quartile, third quartile], depending on their normality assessed with the Shapiro–Wilk test. These variables were compared using Student’s *t*-test or U-Mann–Whitney, if appropriate. Categorical variables are presented as counts and percentages. Intergroup differences were assessed via Pearson’s chi-squared or Fisher’s exact tests.

To determine correlations between echocardiographic measurements (AVA, LVMI, aortic peak velocity, mean transaortic pressure gradient (Pmean), and Pmax) and ECG tracings that are characteristic for LVH, including Sokolov–Lyon and Cornell criteria, Spearman’s rank correlation test was introduced.

All factors, including clinical characteristics and CAD status, that may have been associated with LVH confirmed by ECG and echocardiography were included in univariate regression models. Those that reached a statistical significance of *p* < 0.2 were consecutively adopted in the multivariate regression models. Risk estimates were presented as odds ratio (OR), with 95% confidence intervals (CI). *p* value < 0.05 was adopted as statistically significant. The analysis was carried out with the use of Statistica version 13.3 (TIBCO Software, Palo Alto, CA, USA).

## 3. Results

### 3.1. General Characteristics at Baseline and Clinical Presentation

A total of 74 patients (36 males) with a mean age of 77.9 (± 9.4) were included in this study. Echocardiographic LVH was present in 49 patients (66.2%), whereas ECG-LVH was present in 35 (47.3%) patients. Twenty-seven (36.5%) patients had LVH confirmed in both echocardiography and ECG. As shown in Table 1, the patients’ demographic and clinical characteristics at baseline were similar between the study groups. Moreover, presented symptoms, advancement of CAD (expressed as the number of vessels with significant stenoses), as well as interventional treatment choice (SAVR vs. TAVI) were also comparable (Table 2). Only the CCS class distribution differed between the study groups and reached statistical significance—patients with LVH confirmed in echocardiography more often had CCS classes ≥ III (*p* = 0.04, Table 2).

### 3.2. Pharmacology

The majority of the studied population had taken beta-blockers (81.1%), statins (62.62%), and diuretics (60.8%). No significant differences were observed in terms of medication. More detailed data are shown in Table 3.

### 3.3. ECG and Echocardiographic Findings

Importantly, there were no statistically significant differences found between the prevalence of ECG-LVH (Cornell or Sokolov–Lyon criterium) in the study groups (Table 4). Other ECG tracings that are characteristic for LVH were also comparable, with the exception of the R wave’s amplitude being higher or equal to 1.1 mV in aVL and exceeding 2.6 mV in V5 or V6. These two criteria were significantly more prevalent in patients with ECHO-LVH (32.7% vs. 12% and 18.4% vs. 0%, respectively, Table 4).

Considering the echocardiographic findings, the median LVMI equaled 136 [116; 156] g/m^2^ in the ECHO-LVH group and 89 [78; 95] g/m^2^ in the group without ECHO-LVH (Table 5). Furthermore, the composites of LVMI, i.e., posterior wall thickness at end-diastole (PWd) and interventricular septal thickness at end-diastole (IVSd), were also significantly greater among patients with LVH-ECHO. However, other echocardiographic measurements, i.e., AVA, Pmax, Pmean, and Vmax, did not differ significantly between the compared groups. LVEF was normal (60 [50; 60], Table 5).

### 3.4. ECG Criteria as Diagnostic Tests for Echocardiography-Dependent LVH

Overall, the sensitivity of the ECG criteria for LVH recognition was low, varying in the overall population from 16.3% in the criterium concerning the sum of the RI and SIII waves amplitudes exceeding 2.5 mV to 40.8% in both the Sokolov–Lyon and Cornell criteria (Table 6). The latter two yielded overall similar results. What is more, the positive predictive values were greater than the negative predictive values in all circumstances. 

When considering patients with SVD only, the ECG criterium of the RI wave’s amplitude being higher or equal to 1.2 mV reached the highest sensitivity, i.e., 62.5%, with an accuracy of 76.9%. What is more, the sensitivity was higher among patients with SVD compared to those with MVD in almost all criteria. The only exception was the LVH-ECG trace of an R wave’s amplitude in aVL being higher or equal to 1.1 mV.

### 3.5. Correlation between ECG Criteria That are Characteristic for LVH and Echocardiographic Measurements

For different ECG criteria, significant correlations were rare and found only with LVMI measurements (Table 7). These correlations were weak, reaching the highest value for the McPhil criterium (Rmax + Smax in precordial leads being higher or equal to 4.5 mV) (R = 0.41, *p* < 0.001). Sokolow–Lyon and Cornell indices were comparable in correlation to LVMI (R = 0.31, *p* = 0.008, and R = 0.32, respectively; *p* = 0.005).

### 3.6. Factors Associated with ECG-Dependent and Echocardiographic-Dependent LVH

Univariate and multivariate analyses for ECG-LVH and echocardiographic LVH are summarized in Table 8 and Table 9. Although the LVMI was associated with ECG-LVH (OR: 1.02; 95% CI: 1.00–1.03; *p* = 0.02) in the univariate analysis, it was not confirmed in the multivariate model. Moreover, no other echocardiographic parameters were significantly linked to ECG-LVH, nor to angina symptoms and coronary vessel status (i.e., significant stenoses prevalence and number of vessels involved in CAD). However, CCS class I presence, compared to CCS class 0, was associated with a significantly higher chance for ECG-LVH. Similar results were obtained in logistic regression models for LVH and confirmed by echocardiography.

## 4. Discussion

Although multiple studies have already aimed at describing the concordance between ECHO- and ECG-LVH, there are still scarce data regarding its recognition in light of the concomitant presence of significant stenoses in the coronary arteries. To the best of our knowledge, this analysis is one of the few that provides insight into this issue. Our major findings are as follows: (i) the ECG criteria for ECHO-LVH detection achieved only low sensitivity, which did not differ greatly when considering patients with SVD and MVD separately; (ii) correlations between ECG-LVH changes and echocardiographic measurements were present for LVMI, but not for hemodynamic status, and were overall weak; (iii) neither the location of significant stenoses in coronary arteries nor the advancement of significant CAD were confirmed as factors that are associated with either ECG- or ECHO-LVH.

In general, a natural progression of AS exhibits a two-phase pattern, featuring an extended asymptomatic pre-clinical stage, succeeded by a marked decline in later stages as the initially adaptive processes of LVH and fibrosis transition into systolic and diastolic heart failure [6,16,17]. However, even among patients with severe AS, symptoms may be absent in almost one-fourth of patients [18]. That, in turn, impacts the treatment choice, as present recommendations advocate for SAVR for individuals who are diagnosed with severe AS and who display symptomatic indications or left ventricular systolic impairment, as indicated by a decreased LVEF [19]. On the other hand, TAVI is a potentially attractive treatment option for AS given its expeditious and less invasive characteristics, and it has become an established treatment option for patients with symptomatic AS and is supported by class IA recommendations in current European and US guidelines on the management of valvular heart diseases [20]. In our analysis, the prevalence of SAVR vs. TAVI did not differ between patients with ECHO-LVH with and without ECG-LVH and those without echocardiographic LVH.

In our study, only 55% of patients with echocardiographic LVH met the ECG criteria for LVH recognition. This discordance, which is well reported by other authors, suggests complex underlying mechanisms differentiating so-called electrical and anatomical LVH, which should be portrayed as a distinctive entity [7,9,21]. The vast majority of ECG criteria rely on the QRS complex voltage, which is altered predominantly by the left ventricular mass and its shape. Hence, ECG might not fully encompass the complexity of other myocardial changes that are secondary to LVH, i.e., myocardial dysfunction, interstitial fibrosis, and changes at the level of cardiomyocytes [7]. On the other hand, other subsidiary factors may significantly impact ECG recordings, such as the conduction velocity, myocardial tension, and biochemical changes [7]. Consequently, the feasibility of ECG detecting LVH varies among patients based on their clinical history, including CAD. For instance, prior MI resulting in scar tissue from the infarcted area can alter the electrical conduction pathways in the heart, potentially leading to changes in the ECG patterns that are associated with LVH and, overall, lowered sensitivity.

When testing ECG’s diagnostic ability to detect LVH in the light of ECHO, we achieved high specificity (up to 100%) with poor sensitivity. Similar results were obtained regardless of the advancement of obstructive CAD. The best sensitivity was obtained for the Sokolov–Lyon and Cornell criteria, in which it barely exceeded 40%. Poor diagnostic characteristics were reported in multiple other studies, often with a sensitivity lower than 25% [7,22,23,24]. What is more, the superiority of the Cornell voltage criteria was also frequently observed—Sjoberg et al. reported a sensitivity level of 50% [8,25,26]. It may be the case that the Cornell criterium advantage is driven by the fact that it encompasses both the frontal and horizontal heart planes, whereas the majority of other ECG tracings rely solely on one plane. In our case, the Cornell and Sokolov–Lyon criteria yielded the same results. 

When it comes to other diagnostic test characteristics, the positive predictive value was greater than the negative predictive value in all evaluated criteria. This can be explained simply by the high prevalence of ECHO-LVH in all compared subgroups, i.e., total population (66.2%), patients with SVD (61.5%), MVD (87.5%), and those without significant stenoses (69.5%). 

Due to the low sensitivity and discrepancies between ECHO- and ECG-LVH, an electrocardiogram as a diagnostic tool for LVH remains unsatisfactory. However, given the fact that ECG captures different aspects of LVH, it brings an unneglectable clinical value, such as the prognostic ability for an increased risk of ventricular and atrial arrhythmias, sudden cardiac death, and overall cardiovascular morbidity [7,9,27,28]. Importantly, these associations remained significant independently of LVH, confirmed either through cardiac MRI or echocardiography. What is more, in the light of varying CAD advancement among patients with AS, ECG was proven to have an important clinical value while referring to TAVI. It has been revealed that the presence of preprocedural ECG-LVH is associated with better outcomes following TAVI intervention, including lower risks of major adverse cardiac and cerebrovascular effects (MACCE) [29,30,31]. At the same time, the presence of LVH according to echocardiography is associated with poorer outcomes following TAVI, including a higher death rate at 5-year follow-up [32,33]. Yang et al. discussed that surprising finding in detail, suggesting that ECG-LVH may reflect greater reverse remodeling after TAVI or a lack of extensive preprocedural fibrosis [31]. That indicates the ongoing clinical usage of the traditional ECG for LVH characterization among patients with severe AS and different hemodynamic status, despite a poor diagnostic ability to detect echocardiographic LVH. 

The associations between ECG criteria and echocardiographic measurements have been extensively studied before, and they vary greatly depending on the studied cohort. In our paper, correlations between LVMI and multiple ECG-LVH criteria were observed to be weak and already well recognized in the literature [8,25,31,34]. However, the multivariate analysis did not confirm ECG traces as a predictor of echocardiographic LVH. What is more, no correlations were noted between ECG-LVH traces and echocardiographic markers of AS severity (AVA, Pmean, Pmax, and Vmax), which is inconsistent with other findings. In general, patients with LVH confirmed by ECG have rather more severe AS hemodynamics; however, results obtained by other authors vary considerably [8,31,35]. For instance, Bula et al. revealed only weak to moderate correlations between selected ECG criteria and AS–echocardiographic severity indices. The strongest correlation was reported for the Sokolov–Lyon criterium and peak aortic jet velocity (r = 0.49) [25]. A similar association was found by Greve et al., who studied only asymptomatic patients with AS [35]. Budkiewicz et al. reported positive associations between the peak transaortic pressure gradient and the Sokolov–Lyon and Rimhilt voltage criteria [8]. Importantly, these remained significant after the adjustment for LVMI, age, and BMI. The lack of statistically significant correlations in our study might have been driven by an insufficient sample size, as well as several factors differentiating our population, including the fact that prior MI was relatively frequent in our study group. Nevertheless, the body of evidence reported by other papers adds to the aforementioned premise, suggesting that ECG assessment for AS-derived LVH traces should not be omitted, as it might provide additional insight into the patient’s clinical condition and further prognosis.

As a part of patients’ management and following treatment choice, our study group underwent coronary angiography to assess the prevalence of significant stenoses. An association between CAD and severe AS is well reported in the literature. This is driven by the similarity of the pathogenesis behind these two pathologies: phenomena that are characteristic for atherosclerosis, i.e., lipid deposition and inflammatory cell infiltration, also figure prominently in the early phase of aortic degeneration [36,37]. Moreover, this association is marked by shared risk factors, including smoking and hypertension. Hence, it could be hypothesized that patients’ CAD advancement might be associated with ECHO- and ECG-LVH indices, and that it may come in handy in patient stratification according to ECG’s feasibility for echocardiography LVH recognition. However, our study does not support this hypothesis, since the advancement of CAD (SVD vs. MVD vs. no significant stenoses) did not considerably impact the diagnostic feasibility of ECG and was linked neither to ECHO-LVH nor ECG-LVH. Therefore, this analysis confirms the insufficient sensitivity of ECG in ECHO-LVH detection. Nevertheless, taking into account the existing body of evidence regarding other values brought by classical ECG, both tests should be used in clinical practice among patients with severe AS, regardless of the CAD status.

Further studies encompassing larger populations and more detailed data on hemodynamic status are necessary to provide a valuable consensus on MVD’s and SVD’s associations to ECHO- and ECG-LVH features. Moreover, instead of concentrating solely on significant stenoses that are assessed via coronary angiography, it might be worth exploring multimodal assessment of CAD with the use of advanced echocardiography. New indices, such as global longitudinal strain and mechanical dispersion, were shown to increase the accuracy of obstructive CAD detection [38,39]. Moreover, the assessment of myocardial strain has a well-validated prognostic value [40].

### Limitations

This study is limited by several factors. Firstly, the population was small, including the fact that only a part of the population had at least one significant stenosis in the coronary artery. This impacted the statistical power and reliability of the obtained results. Similarly, the results of multivariate analysis are not precise and might be faulty due to the small sample. Moreover, we lacked data regarding other hemodynamic parameters, such as the coronary and fractional flow reserve, to further study CAD characteristics. Furthermore, due to the lack of blinding, there could have been a bias in ECG record assessments.

## 5. Conclusions

ECG criteria for ECHO-LVH detection achieved only low sensitivity, which did not differ greatly when considering patients without significant stenosis, with single-, and multi-vessel disease considered separately. A correlation between ECG-LVH traces and echocardiographic measurements was present only for LVMI and was weak. Lastly, neither the location of coronary arteries’ significant stenoses nor their distribution (single- vs. multi-vessel disease) were confirmed as factors that are associated with ECG- or ECHO-LVH.

## Figures and Tables

**Table 1:** Patients’ demographic and clinical characteristics.

Variable	Total N = 74	ECHO-LVH		*p* Value
		Yes N = 49	No N = 25	
Mean age ± SD, years	77.9 ± 9.4	77.5 ± 9.2	78.8 ± 9.9	0.58
Gender, male	36 (48.6)	27 (55.1)	14 (56.0)	0.37
Obesity (BMI ≥ 30)	20 (27.0)	12 (24.5)	8 (32.0)	0.49
Diabetes mellitus	30 (40.5)	19 (38.8)	11 (44.0)	0.67
Prior MI	14 (18.9)	9 (18.4)	5 (20.0)	0.87
Smoking history	18 (24.3)	11 (22.5)	7 (28.0)	0.60
Current smoker	5 (6.8)	3 (6.1)	2 (8.0)	0.76
Arterial hypertension	64 (86.5)	44 (89.8)	20 (80.0)	0.24
CKD	11 (14.9)	5 (10.2)	6 (24.0)	0.10
CAD	49 (66.2)	33 (67.3)	16 (64.0)	0.66
Hyperthyroidism	2 (2.7)	2 (4.1)	0 (0.0)	0.62
Hypothyroidism	8 (10.8)	6 (12.2)	2 (8.0)	0.32
AF	18 (24.3)	12 (24.5)	6 (24.0)	0.96
Hyperlipidaemia	41 (55.4)	26 (53.1)	15 (60.0)	0.57
Heart failure	34 (45.9)	26 (53.1)	8 (32.0)	0.08
ECG-LVH	35 (47.3)	27 (55.1)	8 (32.0)	0.06

All data are expressed as absolute numbers (percentages), if not stated otherwise. AF, Atrial fibrillation; BMI, body mass index; CAD, coronary artery disease; CKD, chronic kidney disease; ECG, electrocardiogram; ECHO, echocardiography; MI, myocardial infarction; LVH, left ventricular hypertrophy; SD, standard deviation.

**Table 2:** Clinical presentation, hemodynamic status, and treatment choice.

Variable	Total N = 74	ECHO-LVH		*p* Value
		Yes N = 49	No N = 25	
CCS class				0.04
I	13 (17.6)	9 (18.4)	4 (16.0)	
II	17 (23.0)	7 (14.3)	10 (40.0)	
III	8 (10.8)	8 (16.3)	0 (0.0)	
IV	1 (1.4)	1 (2.0)	0 (0.0)	
None	15 (20.3)	11 (22.4)	4 (0.0)	
NYHA class				0.16
I	5 (6.8)	3 (6.1)	2 (8.0)	
II	24 (32.4)	12 (24.5)	12 (48.0)	
III	20 (27.0)	17 (34.7)	3 (12.0)	
IV	5 (6.8)	4 (8.2)	1 (4.0)	
None	20 (27.0)	6 (12.2)	7 (28.0)	
Shortness of breath	37 (50.0)	26 (53.1)	11 (44.0)	0.31
Chest pain ^a^	18 (24.3)	14 (28.6)	4 (16.0)	0.23
Sudden cardiac arrest	3 (4.1)	3 (6.1)	0 (0.0)	0.21
Syncope episode	6 (8.1)	4 (8.2)	2 (8.0)	0.98
Significant stenosis within LMCA	6 (8.1)	5 (10.2)	1 (4.0)	0.43
CAD advancement				0.61
Single-vessel disease	13 (17.6)	8 (16.3)	5 (20.0)	
Double-vessel disease	6 (8.1)	5 (10.2)	1 (4.0)	
Triple-vessel disease	2 (2.7)	2 (4.1)	0 (0.0)	
Treatment choice for AS				0.36
SAVR	26 (35.1)	19 (38.8)	7 (28.0)	
TAVI	40 (54.1)	26 (53.1)	14 (56.0)	
Conservative treatment	3 (4.1)	1 (2.0)	2 (8.0)	

All data are expressed as absolute numbers (percentages). ^a^ Pain on exertion and at rest was taken into account. AS, aortic stenosis; CAD, coronary artery disease; CCS, Canadian Cardiovascular Society; ECHO, echocardiography; LMCA, left main coronary artery; LVH, left ventricular hypertrophy; NYHA, New York Heart Association; TAVI, transcatheter aortic valve implantation; SAVR, surgical aortic valve replacement/repair.

**Table 3:** Pharmacotherapy.

Variable	Total N = 74	ECHO-LVH		*p* Value
		Yes N = 49	No N = 25	
BB	60 (81.1)	38 (77.6)	22 (88.0)	0.30
CCB	17 (23.0)	12 (24.5)	5 (20.0)	0.44
ACEI	28 (37.8)	19 (38.8)	9 (36.0)	0.59
ARB	18 (24.3)	13 (26.5)	5 (20.0)	0.98
Statin	46 (62.2)	33 (67.3)	13 (52.0)	0.89
Diuretics	45 (60.8)	31 (63.3)	14 (56.0)	0.36
Antiarrhythmic	2 (2.7)	1 (2.0)	1 (4.0)	0.33
Oral Hypoglycaemic medications	18 (24.3)	11 (22.4)	7 (28.0)	0.49
Insulin	6 (8.1)	2 (4.1)	4 (16.0)	0.09
ASA	36 (48.6)	23 (46.9)	13 (52.0)	0.35
PPI	25 (33.8)	18 (36.7)	7 (28.0)	0.71
NOAC/OAC	16 (21.6)	11 (22.4)	5 (20.0)	0.93

All data are expressed as absolute numbers (percentages). ACEI, angiotensin-converting enzyme inhibitors; ARB, Angiotensin receptor blockers; ASA, acetylsalicylic acid; BB, beta-blockers; CCB, calcium channel blockers; NOAC, non-vitamin K antagonist oral anticoagulants; OAC, oral anticoagulants; PPI, proton pump inhibitors.

**Table 4:** Electrocardiogram characteristics and criteria for left ventricular hypertrophy.

Variable	Total N = 74	ECHO-LVH		*p* Value
		Yes N = 49	No N = 25	
R in I ≥ 12 mm	24 (32.4)	19 (38.8)	5 (20.0)	0.10
R in aVL ≥ 11 mm	19 (25.7)	16 (32.7)	3 (12.0)	0.05
R in aVF ≥ 20 mm	1 (1.4)	1 (2.0)	0 (0.0)	0.47
R I + S III > 25 mm	11 (14.9)	8 (16.3)	3 (12.0)	0.62
R max + S max (of the V1–V6) ≥ 45 mm	12 (16.2)	10 (20.4)	2 (8.0)	0.12
R in V5 or V6 > 26 mm	9 (12.2)	9 (18.4)	0 (0.0)	0.02
Sokolov-Lyon criterium	25 (33.8)	20 (40.8)	5 (20.0)	0.07
Cornell criterium	25 (33.8)	20 (40.8)	5 (20.0)	0.07
Left axis deviation	19 (25.7)	12 (24.5)	7 (28.0)	0.71
T wave in V5 or V6				0.16
Positive	49 (66.2)	30 (61.2)	20 (80.0)	
Negative	19 (25.7)	16 (32.7)	3 (12.0)	
Biphasic	5 (6.8)	3 (6.1)	2 (8.0)	
ST segment				0.86
Normal	60 (81.1)	41 (83.7)	21 (84.0)	
Elevated	2 (2.7)	1 (2.0)	1 (4.0)	
Depressed	10 (13.5)	7 (14.3)	3 (12.0)	

All data are expressed as absolute numbers (percentages). ECHO-LVH, echocardiographic left ventricular hypertrophy.

**Table 5:** Echocardiographic characteristics.

Variable	Total N = 74	ECHO-LVH		*p* Value
		Yes N = 49	No N = 25	
LVEF, %	60 [50; 60]	60 [45; 60]	60 [53; 65]	0.19
IVS thickness, mm	13 [11; 14]	13 [11; 14]	12 [11; 13]	0.02
PW thickness, mm	11 [10; 12]	11 [10; 13]	10 [9; 11]	<0.001
AVA, cm^2^	0.60 [0.55; 0.70]	0.7 [0.6; 0.8]	0.6 [0.5; 0.7]	0.17
Pmax, mmHg	77.2 [68.6; 103.0]	82.1 [70.5; 103.8]	75.9 [65.3; 83.0]	0.20
Pmean, mmHg	49.3 [42.0; 60.5]	51.1 [42.5; 64.5]	45.5 [41.7; 52.3]	0.19
Vmax, m/s	4.4 [4.1; 5.0]	4.5 [4.2; 5.1]	4.4 [4.0; 4.6]	0.23
LVMI, g/m^2^	116.60 [95.65; 144.50]	136.2 [116.0; 156.0]	88.9 [78.0; 95.3]	<0.001

All data are expressed as absolute numbers (percentages) if not stated otherwise. AVA, aortic valve area; IVS, interventricular Septum; LVEF, left ventricle ejection fraction; LVH, left ventricular hypertrophy; LVMI, left ventricular mass index; Pmax, maximal transaortic pressure gradient; Pmean, mean transaortic pressure gradient; PW, posterior wall; Vmax, peak velocity.

**Table 6:** Diagnostic test characteristics for ECG-LVH criteria confirmed by echocardiography–LVH.

Variable	Group ^a^	Sensitivity, %	Specificity, %	PPV, %	NPV, %	Accuracy, %
R in I ≥ 12 mm	Total (n = 74)	38.8	80.0	79.2	40.0	52.7
	SVD (n = 13)	62.5	100	100	62.5	76.9
	MVD (n = 8)	57.1	100	100	25.0	62.5
	No significant CAD (n = 46)	31.5	64.3	66.7	29.0	41.3
R in aVL ≥ 11 mm	Total (n = 74)	32.7	88.0	84.2	40.0	51.4
	SVD (n = 13)	37.5	100	100	50.0	61.5
	MVD (n = 8)	28.6	100	100	16.7	37.5
	No significant CAD (n = 46)	34.8	85.7	84.6	36.3	50.0
R I + S III > 25 mm	Total (n = 74)	16.3	88.0	72.7	34.9	40.5
	SVD (n = 13)	12.5	100	100	41.8	46.2
	MVD (n = 8)	14.3	100	100	14.3	25.0
	No significant CAD (n = 46)	18.8	85.7	75.0	31.5	39.1
R max + S max (of the V1–V6) ≥ 45 mm	Total (n = 74)	24.4	91.3	84.6	38.1	48.4
	SVD (n = 13)	16.7	100	100	42.9	54.6
	MVD (n = 8)	-				
	No significant CAD (n = 46)	28.6	91.7	88.7	35.9	47.5
R in V5 or V6 > 26 mm	Total (n = 74)	18.8	100	100	38.6	46.6
	SVD (n = 13)	25.0	100	100	45.5	53.9
	MVD (n = 8)	14.3	100	100	14/3	25.0
	No significant CAD (n = 46)	19.4	100	100	35.1	44.4
Sokolov–Lyon	Total (n = 74)	40.8	80.0	80.0	40.8	54.1
	SVD (n = 13)	50.0	80.0	80.0	50.0	61.5
	MVD (n = 8)	42.9	100	100	20.0	50.0
	No significant CAD (n = 46)	37.5	78.6	80.0	35.5	50.0
Cornell	Total (n = 74)	40.8	80.0	77.3	44.8	54.1
	SVD (n = 13)	37.5	100	100	50.0	61.5
	MVD (n = 8)	-				
	No significant CAD (n = 46)	43.8	71.4	77.7	35.8	52.2

^a^ Patients were allocated into 3 groups regarding their coronary artery disease status and its advancement (No CAD, SVD = single-vessel disease, MVD = multi-vessel disease). In certain cases, there was not a large enough group to define diagnostic test characteristics. CAD, coronary artery disease; ECG-LVH, electrocardiographic left ventricular hypertrophy; NPV, negative predictive value; PPV, positive predictive value.

**Table 7:** Spearman’s rank test for correlation testing between ECG and echocardiographic characteristics for LVH.

Variable	AVA (cm^2^)	LVMI (g/m^2^)	Vmax (m/s)	Pmean (mmHg)	Pmax (mmHg)
R in I ≥ 12 mm	R = 0.01	R = 0.17	R = 0.23	R = 0.11	R = 0.12
	*p* = 0.93	*p* = 0.16	*p* = 0.06	*p* = 0.34	*p* = 0.32
R in aVL ≥ 11 mm	R = 0.11	R = 0.36	R = 0.12	R = 0.09	R = 0.07
	*p* = 0.34	*p* = 0.002	*p* = 0.35	*p* = 0.47	*p* = 0.55
R in aVF ≥ 20 mm	R = −0.1	R = 0.05	R = 0.05	R = 0.003	R = 0.08
	*p* = 0.42	*p* = 0.67	*p* = 0.69	*p* = 0.98	*p* = 0.51
R I + S III > 25 mm	R = 0.05	R = 0.31	R = 0.14	R = 0.1	R = 0.1
	*p* = 0.65	*p* = 0.009	*p* = 0.26	*p* = 0.40	*p* = 0.39
R max + S max (of the V1–V6) ≥ 45 mm	R = −0.1	R = 0.41	R = 0.01	R = 0.04	R = 0.12
	*p* = 0.4	*p* = <0.001	*p* = 0.95	*p* = 0.72	*p* = 0.34
R in V5 or V6 > 26 mm	R = −0.05	R = 0.21	R = −0.03	R = −0.04	R = 0.01
	*p* = 0.69	*p* = 0.07	*p* = 0.82	*p* = 0.74	*p* = 0.92
Sokolow–Lyon	R = −0.13	R = 0.31	R = 0.004	R = 0.06	R = 0.1
	*p* = 0.29	*p* =0.008	*p* = 0.97	*p* = 0.60	*p* = 0.4
Cornell voltage	R = 0.08	R = 0.32	R = 0.12	R = 0.22	R = 0.20
	*p* = 0.5	*p* = 0.005	*p* = 0.37	*p* = 0.07	*p* = 0.10

Abbreviations as in Table 5.

**Table 8:** Uni- and multivariate analyses for the ECG-dependent LVH.

Variable	Univariate			Multivariate		
	OR	95% CI	*p* Value	OR	95% CI	*p* Value
Echocardiographic LVH	2.608	0.948–7.171	0.06	1.930	0.202–18.409	0.57
CKD	0.352	0.085–1.451	0.15	0.642	0.073–5.618	0.69
CAD	0.390	0.136–1.116	0.08	0.245	0.037–1.619	0.14
CCS class I ^a^	6.667	1.244–35.715	0.03	24.967	1.737–358.859	0.02
CCS class II ^b^	1.091	0.252–4.714	0.91	1.805	0.294–11.083	0.52
CCS class III ^c^	3.333	0.557–19.949	0.19	3.414	0.355–32.788	0.29
LVMI	1.017	1.003–1.031	0.02	1.014	0.991–1.037	0.24
AVA	2.897	0.206–40.717	0.43	-		
Aortic Peak Velocity	0.769	0.433–1.365	0.37	-		
Pmean	0.998	0.979–1.018	0.85	-		
Significant stenosis in LAD	0.621	0.136–2.837	0.54	-		
Significant stenosis in Cx	0.802	0.165–3.893	0.78	-		
Significant stenosis in RCA	1.385	0.378–5.070	0.62	-		
Significant stenosis in LMCA	0.517	0.088–3.034	0.46	-		
Single-vessel disease	0.625	0.178–2.199	0.46	-		
Multi-vessel disease	1.000	0.223–4.489	1.00	-		
Multi-vessel vs. single-vessel disease	1.600	0.270–9.490	0.60	-		
Shortness of breath	1.020	0.398–2.617	0.97	-		
Chest pain	1.550	0.532–4.513	0.42	-		

^a,b,c^ As compared to CCS class 0; AVA, aortic valve area; CAD, coronary artery disease; CKD, chronic kidney disease; Cx, circumflex (artery); LAD, left anterior descending (artery); LMCA, left main coronary artery; LVH, left ventricular hypertrophy; LVMI, left ventricular mass index; RCA, right coronary artery.

**Table 9:** Uni- and multivariate analyses for LVH confirmed by echocardiography.

Variable	Univariate			Multivariate		
	OR	95% CI	*p* Value	OR	95% CI	*p* Value
Significant stenosis in LAD	3.325	0.381–28.985	0.28	-		
Significant stenosis in Cx	1.071	0.190–6.045	0.94	-		
Significant stenosis in RCA	1.162	0.274–4.926	0.84	-		
Significant stenosis in LMCA	2.262	0.247–20.710	0.47	-		
Single-vessel disease	0.615	0.173–2.183	0.45	-		
Multi-vessel disease	0.327	0.037–2.910	0.32	-		
Multi- vs. single-vessel disease	4.375	0.407–47.019	0.22	-		
R I + S III > 25 mm	1.070	1.003–1.141	0.04	-		
R in aVL > 11 mm	1.154	1.015–1.310	0.03	-		
R in aVF > 20 mm	0.995	0.866–1.144	0.94	-		
Cornell criterium	1.031	0.980–1.084	0.24	-		
Sokolov–Lyon criterium	1.041	0.995–1.089	0.08	0.281	0.005–15.861	0.54
LVEF (%)	0.963	0.921–1.008	0.10	0.991	0.055–17.778	0.99
ECG-LVH	2.608	0.948–7.171	0.06	-		
Chest pain	2.100	0.610–7.227	0.24	-		
Shortness of breath	1.351	0.497–3.672	0.56	-		

Cx, circumflex (artery); LAD, left anterior descending (artery); LMCA, left main coronary artery; LVEF, left ventricle ejection fraction; LVH, left ventricular hypertrophy; RCA, right coronary artery.

## Data Availability

Data can be made available upon special request.
